# Dysosteosclerosis: Clinical and Radiological Evolution Reflecting Genetic Heterogeneity

**DOI:** 10.1002/jbm4.10663

**Published:** 2022-07-28

**Authors:** Serap Turan, Steven Mumm, Ceren Alavanda, Betul Sare Kaygusuz, Busra Gurpinar Tosun, Ahmet Arman, Margaret Huskey, Tulay Guran, Shenghui Duan, Abdullah Bereket, Michael P. Whyte

**Affiliations:** ^1^ Pediatric Endocrinology and Diabetes Marmara University School of Medicine Başıbüyük Mah. Maltepe Başıbüyük Yolu Sok. No:9/2 Istanbul 34854 Türkiye; ^2^ Division of Bone and Mineral Diseases, Department of Internal Medicine Washington University School of Medicine St. Louis MO USA; ^3^ Center for Metabolic Bone Disease and Molecular Research Shriners Hospitals for Children St. Louis MO USA; ^4^ Medical Genetics Marmara University Faculty of Medicine Istanbul Turkey

**Keywords:** BONE TURNOVER, COLONY STIMULATING FACTOR, METAPHYSEAL SCLEROSIS, OSTEOCALCIN, OSTEOPETROSIS, OSTEOSCLEROSIS, PYLE DISEASE, SKELETAL DYSPLASIA, *SLC29A3*, *TCIRG1*, *TNFRSF11A*, *CSF1R*, FRACTURES, RANK, RANKL, RECEPTOR ACTIVATOR OF NUCLEAR FACTOR ΚB, DEVELOPMENTAL DELAY, DUAL‐ENERGY X‐RAY ABSORPTIOMETRY, HYPERCALCEMIA, *LRRK1*, METABOLIC BONE DISEASE, METAPHYSEAL DYSPLASIA

## Abstract

Dysosteosclerosis (DSS), the term coined in 1968 for ultrarare dysplasia of the skeleton featuring platyspondyly with focal appendicular osteosclerosis, has become generic by encompassing the genetic heterogeneity recently reported for this phenotype. We studied four unrelated Turkish patients with DSS to advance understanding of the new nosology. Patient 1 suffered femur fractures beginning at age 1 year. DSS was suspected from marked metaphyseal osteosclerosis in early childhood and subsequently platyspondyly accompanying patchy osteosclerosis of her appendicular skeleton. She harbored in *SLC29A3*, in 2012 the first gene associated with DSS, a unique homozygous duplication (c.303_320dup, p.102_107dupYFESYL). Patient 2 presented similarly with fractures and metaphyseal osteosclerosis but with no platyspondyly at age 2 months. She was homozygous for a novel nonsense mutation in *SLC29A3* (c.1284C>G, p.Tyr428*). Patient 3 had ocular disease at age 2 years, presented for short stature at age 11 years, and did not begin to fracture until age 16 years. Radiographs showed mild platyspondyly and focal metaphyseal and femoral osteosclerosis. She was homozygous for a unique splice site mutation in *TNFRSF11A* (c.616+3A>G). Patient 4 at age 2 years manifested developmental delay and frequent infections but did not fracture. He had unique metadiaphyseal splaying and osteosclerosis, vertebral end‐plate osteosclerosis, and cortical thinning of long bones but no mutation was detected of *SLC29A3*, *TNFRSF11A*, *TCIRG1*, *LRRK1*, or *CSF1R* associated with DSS. We find that DSS from defective *SLC29A3* presents earliest and with fractures. DSS from compromised *TNFRSF11A* can lead to optic atrophy as an early finding. Negative mutation analysis in patient 4 suggests further genetic heterogeneity underlying the skeletal phenotype of DSS. © 2022 The Authors. *JBMR Plus* published by Wiley Periodicals LLC on behalf of American Society for Bone and Mineral Research.

## Introduction

Dysosteosclerosis (DSS or DOS), considered an osteopetrosis (OPT) when first described in 1934 by Ellis,^(^ ^1^ ^)^ was named in 1968 by Spranger and colleagues^(^ ^2^ ^)^ to emphasize its patchy osteosclerosis^(^ ^1^, ^2^ ^)^ accompanied by platyspondyly with vertebral endplate thickening (OMIM % 224300).^(^ ^3^ ^)^ During infancy and early childhood the osteosclerosis of DSS is widely distributed but especially pronounced in expanded metaphyses, whereas diaphyses are broad and radiodense or radiolucent.^(^ ^2^, ^3^, ^4^, ^5^, ^6^, ^7^, ^8^, ^9^, ^10^ ^)^ Then, these features of DSS evolve.^(^ ^4^, ^5^, ^6^, ^10^ ^)^ Expanded osteosclerotic metaphyses become osteopenic, sometimes including portions of the diaphyses where the cortex may be thin.^(^ ^4^, ^6^, ^10^ ^)^ Cranial nerve palsies and developmental delay can occur.^(^ ^6^, ^7^, ^8^, ^9^, ^10^ ^)^ Skin changes are sometimes noted.^(^ ^1^, ^4^, ^7^ ^)^ By middle age, there can be diffuse osteosclerosis of cranial bones, mandible, maxilla, mastoid, and skull base.^(^ ^6^, ^10^, ^11^ ^)^ Diagnosing DSS may be delayed because its radiographic hallmarks can go undocumented or misunderstood. In 2010, we reported that DSS in early childhoodrepresents an “osteoclast‐poor” form of OPT^(^ ^4^ ^)^ wherein some heritable defect transiently abrogates osteoclastogenesis.^(^ ^4^, ^5^ ^)^

Delineation of the etiology of DSS began in 2012 when we reported two girls who harbored biallelic mutations of the nucleoside transporter gene “solute carrier family 29 member 3” (*SLC29A3*).^(^ ^5^ ^)^ The following year, Sule and colleagues^(^ ^12^ ^)^ found no *SLC29A3* defect in one patient,^(^ ^6^, ^10^ ^)^ thereby suggesting genetic heterogeneity for DSS. In 2016, Iida and colleagues^(^ ^13^ ^)^ associated “leucine rich repeat kinase 1” (*LRRK1*) with osteosclerotic metaphyseal dysplasia (OMD; OMIM % 615198)^(^ ^3^ ^)^ featuring characteristics of DSS.^(^ ^3^, ^13^, ^14^, ^15^, ^16^, ^17^ ^)^ In 2018, two genes underlying autosomal recessive OPT^(^ ^18^, ^19^ ^)^ became associated with DSS^(^ ^20^, ^21^ ^)^: (i) “tumor necrosis factor receptor superfamily member 11A” (*TNFRSF11A*) that encodes receptor activator of NF‐κB (RANK) (OMIM # 612301),^(^ ^3^ ^)^ and (ii) “T‐cell immune regulator 1” (*TCIRG1*) that encodes a component of the vacuolar proton (H+) pump necessary for osteoclasts (OCs) to produce hydrochloric acid (OMIM # 259700).^(^ ^3^ ^)^ Most recently, in 2019, biallelic mutations of “colony‐stimulating factor 1 receptor” (*CSF1R*)^(^ ^22^ ^)^ were associated with dysosteosclerosis‐Pyle disease (DSS‐PD), also called “brain abnormalities, neurodegeneration, and dysosteosclerosis” (BANDDOS: OMIM # 618476).^(^ ^3^ ^)^

Herein, we characterize the phenotype of four young unrelated Turkish patients with DSS. Two carried novel homozygous *SLC29A3* defects, one a novel homozygous *TNFRSF11A* change, and one for whom no mutation was found.

## Patients and Methods

### Patients

The principal clinical and radiological features and results from mutation analyses of our four patients are detailed below and summarized in Table 1.

**Table 1:** Genetic, Clinical, and Radiographic Findings of Our Four Patients

Subject	Patient 1	Patient 2	Patient 3	Patient 4
Genetic defect	*SLC29A3* (c.303_320dup)	*SLC29A3* (c.1284C>G)	*TNFRSF11A* (c.616+3A>G)	None identified
Parental consanguinity	+	+	+	−
Sex	Female	Female	Female	Male
Age at presentation	4 months	2 months	10.6 years	27 months
Complication	Anemia	Fracture	Short stature	Hypercalcemia
Age last visit	23 years	6 months	21 years	4.5 years
Birth weight (gestational age)	4 kg (term)	3270 g (term)	3010 g (term)	2200 g (36 weeks)
Height (SD)	146.3 cm (−2.8 SD)^b^ (final)	62.5 cm (−1.1)	138 cm (−4.2 SD)^b^ (final)	90 cm (−3.8)
Target height (SD)	156 cm (−1.21)	158 cm (−0.87)	154 cm (−1.55)	173 cm (−0.52)
Age of first fracture (# of fractures)	12 months (>7)	2 months (1)	17 years (4)	–
Skin changes	Hypertrichosis	Hypertrichosis	None	Eczema
Optic atrophy	−	−	+	−
Developmental delay	−	−	−	Severe
Recurrent infections	−	−	−	+
Dental problems	Delayed shedding of deciduous teeth	NA	Frequent carries	Discoloration
Radiographs				
Platyspondyly	+	−	+	−
Erlenmeyer flask deformity	+	−	+	+
Diaphyseal osteopenia and thin cortices	+	+	+	+
Osteosclerosis				
Vertebral	+	−	+	+
Rib	−^a^	Mild	+	+
Pelvic bone	+	+	+	+
Calvariae	Mild	−	+	−
Metaphyseal	+	+	−^a^	+
Diaphyseal	+	−	Few and far between	−
Diaphyseal focal sclerosis (femur)	−	−	+	−
Changing with age	+	+	+	+
Hand and forearm bones	+	+	−	+

Abbreviation: NA, not applicable.

^a^ Radiographs taken in older ages, osteosclerosis earlier not excluded.

^b^ Short trunk dwarfism.

#### Patient 1

Patient 1, 23 years‐of‐age, was homozygous for a novel defect in *SLC29A3*. In 2015, we briefly reported her features of DSS and unique mutation.^(^ ^23^ ^)^ Her parents were first‐degree cousins. At age 4 months, severe anemia (hemoglobin [Hb] 3 mg/dL) was attributed to OPT despite no hemolysis or extramedullary hematopoiesis. Subsequently, folic acid supplementation (serum folate 2.9 ng/mL, normal [Nl] 2.0–9.0) corrected this problem. Fracturing began at about age 1 year. Shedding of her deciduous teeth was delayed. Neurodevelopmental milestones were normal with good grades at school. Four of at least seven femoral breaks required surgery, including one to remove metal plates. A supracondylar specimen of femur reportedly showed focal, irregular, lytic, and dysplastic enchondral bone adjacent to mature compact bone. Skeletal scintigraphy at age 12 years revealed symmetrically increased radionuclide uptake attributed to increased osteoblastic activity at the proximal and distal femurs and at the proximal humeral and tibial metadiaphyses. Sequential dual‐energy X‐ray absorptiometry (DXA) demonstrated (Lunar Prodigy; GE Healthcare, Piscataway, NJ, USA) markedly elevated but decreasing areal bone mineral density (aBMD) at the L_1_–L_4_ spine; +10.8 at age 7 years, +6.3 at age 10 years, +6.4 at age 15 years, +8.7 at age 17 years, +7 at age 22 years, and +5.6 at age 23 years. Bone turnover markers (BTMs) spanning ages 11 to 23 years included elevated urinary deoxypyridinoline (DPD; Immulite® 2000, Siemens Healthcare Diagnostic Products Ltd., Llanberis, Gwynedd, UK), but normal serum alkaline phosphatase (ALP; Hitachi 917; Roche Diagnostics International Ltd., Mannheim, Germany), C‐terminal telopeptide (CTX; Cobas e411; Roche Diagnostics International Ltd., Rotkreuz, Switzerland), and procollagen type 1 N‐terminal propeptide (P1NP) (Cobas e411; Roche Diagnostics International Ltd., Rotkreuz, Switzerland). Serum osteocalcin (OCN) levels (Immulite® 2000, Siemens Healthcare Diagnostic Products Ltd., Llanberis, Gwynedd, UK) ranged from low to high (Table 2).

**Table 2:** Genetic, Clinical, and Radiological Spectrum of DSS

Genetic defect (#patient/#family)	*SLC29A3* (6/6)^(^ ^5^, ^21^, ^31^, ^32^ ^)^	*TNFRSF11A* (4/4)^(^ ^11^, ^20^, ^33^, ^34^ ^)^	*TCIRG1* (2/1)^(^ ^21^ ^)^	*CSF1R* (11/5)^(^ ^22^, ^35^, ^36^ ^)^	*LRRK1* (8/5)^(^ ^13^, ^14^, ^15^, ^16^, ^17^ ^)^
Mutations	c.303_320dup (Hom) p.S203P/p.R386Q p.R386Q(Hom) p.P391H (Hom) p.T449R (Hom)	^a^c.616+3A>G (Hom) ^a^c.784G>T (Hom) ^a^c.414**_**427+7del/c.1664del p.R129C (Hom)	c.117+4A>C/p.A796fs*34	p.P132L/p.Q481* p.S620delins40/p.K27del p.P658Sfs*24 (Hom) c.2763+1G>T (Hom) p.Q481* (Het)	p.A34Pfs*33 (Hom) p.E929* (Hom) p.E1980Afs*66 (Hom) p.A1991fs*31 (Hom) p.T1989G1990del (Hom)
Sex ratio	F(6)/M(0)	F(3)/M(1)	F(1)**/**M(1)	F(7)**/**M(4)	F(4)/M(4)
Ethnicity (families)	Turkish(4), ND(1), Cameroonian (1)	Turkish(2), Japanese(1) British (1)	Indian(2)	Brazilian(1), Japanese(1), Chaldean(2+3), Turkish (3), American (1)	Iranian(1), Indian(2), Moroccan(1), Bulgarian(1), Palestinian(3)
Age (years [y] or months [m])	2y, 5y, 5.6y, 22y, 11.1y, 5.5y	16y, 59y, 2.3y, 5.3y	15y, 10y	5y, 35y, 14y, 23y, 9y, 7y, 16y, 14y	14m, 14y, 14y, 23y, 25y, 13.5y, 11.5y, 9m
Short stature	Y(5)**/**N(0)**/**ND(1)	Y(3)**/**N(1)	Y(2)**/**N(0)	Y(2)**/**N(5)/ND(1)	Y(4)**/**N(3)/ND(1)
Skin changes	Y(2)**/**N(4)	Y(0)**/**N(2)/ND(2)	Y(0)**/**N (2)	Y(0)**/**N(0)/ND(8)	Y(0)**/**N(0)/ND(8)
Optic atrophy	Y(0)**/**N(4)**/**ND(2)	Y(3)**/**N(1)	Y(0)**/**N(2)	Y(2)**/**N(3)/ND(3)	Y(2)**/**N(3)/ND(3)
Developmental delay	Y(1)**/**N(5)	Y(1)**/**N(2)/ND(1)	Y(1)**/**N(1?)	Y(8)**/**N(0)	Y(2)**/**N(6)
Recurrent infections	Y(3)**/**N(2)**/**ND(1)	Y^b^(2)/N(1)/ND(1)	Y(0)**/**N(2)	Y(0)**/**N(0)/ND(8)	Y ^b^(2)**/**N(4)/ND(2)
Anemia/pancytopenia	Y(1)**/**N(3)**/**ND(2)	Y(1)**/**N(2)/ND(1)	Y(0)**/**N(2)	Y(0)**/**N(5)/ND(3)	Y(1)**/**N(2)/ND(5)
Hepatosplenomegaly	Y(0)**/**N(2)/ND(4)	Y(1)**/**N(3)	Y(0)**/**N(2)	Y(0)**/**N(7)/ND (1)	Y(1)**/**N(4)/ND(3)
Hypercalcemia	Y(1)**/**N(3)**/**ND(2)	Y(0)**/**N(2)/ND(2)	Y(0)**/**N(2)	Y(0)**/**N(5)/ND(3)	Y(0)**/**N(3)/ND(5)
Dental problems	Y(2)**/**N(4)	Y(3)**/**N(0)/ND(1)	Y(0)**/**N(2)	Y(0)**/**N(0)/ND(8)	Y(5)**/**N(1)/ND(2)
Long bone fractures	Y(5)**/**N(1)	Y(3)**/**N(1)	Y(1) Clavicle**/**N(1)	Y(2)**/**N(6)‐Coccyx	Y(6)**/**N(2)
Delayed fracture healing	Y(1)**/**N(0)**/**ND(5)	Y(0)**/**N(0)/ND(2)/NA(2)	Y(0)**/**ND(1)/NA(1)	Y(0)**/**N(0)/NA(6)/ND(2)	Y(0)**/**N(0)/ND(6)/NA(2)
Radiographs					
Osteosclerosis of calvaria	Mild Y(6)**/**N(0)	Y(4)**/**N(0)	Y(2)**/**N(0)	Y(7)**/**N(0)/ND(1)	Mild Y(2)**/**N(1)/ND(5)
Platyspondyly	Y(6)**/**N(0)	Mild Y(2)**/**Y(2)	Y(2)**/**N(0)	Y(7)**/**N(0)/ND(1)	Y(1)**/**N(4)/ND(3)
Vertebral sclerosis	Y(5)**/**N(0)**/**ND(1)	Y(4)**/**N(0)	Y(2)**/**N(0)	Y(4)**/**N(2)/ND(2)	Y(6)**/**N(0)/ND(2)
Rib sclerosis/thickening	Y(6)**/**N(0)	Y(4)**/**N(0)	Y(2)**/**N(0)	Y(3)**/**N(1?)/ND(4)	Y(4)**/**N(0)/ND(4)
Pelvis‐peripheral sclerosis	Y(3)**/**N(0)**/**ND(3)	Y(2)**/**Diffuse Y(2)/N(0)	Y(0)**/**N(0)**/**ND(2)	Diffuse Y(5)**/**N(2)/ND(1)	Y(4)**/**N(0)/ND(4)
Metaphyseal sclerosis	Y(5)**/**N(0)**/**ND(1)	Y(1)**/**N(3)	Y(2)**/**N(0)	Y(1)**/**N(6)/ND(1)	Y(5)**/**N(3)
Erlenmeyer flask deformity/undertubulation	Y(4)**/**N(2)	Y(3)**/**N(0)/ND(1)	Y(2)**/**N(0)	Y(8)**/**N(0)	Mild Y(6)**/**N(0)/ND(2)
Diaphyseal osteopenia and thin cortices	Y(1)**/**N(5)	Y(2)**/**N(2)	Y(0)**/**N(2)	Y(8)**/**N(0)	Y(3)**/**N(0)/ND(5)
Diaphyseal diffuse sclerosis	Y(0)**/**N(5)**/**ND(1)	Y(2)**/**N(2)	Y(2)**/**N(0)	Y(0)**/**N(8)	Y(0)**/**N(8)
Diaphyseal focal sclerosis	Y(3)**/**N(2)**/**ND(1)	Y(2)**/**N(2)	Y(0)**/**N(2)	Y(8)**/**N(0)	Y(6)**/**N(1)/ND(2)
Changing sclerosis with age	Y(3)**/**N(0)**/**ND(3)	Y(0)**/**N(0)/ND(4)	Y(0)**/**N(0)**/**ND(2)	Y(0)**/**N(0)/ND(8)	Y(2)**/**N(0)/ND(6)
Normal bones of hand and forearm bones	Y(0)**/**N(4)**/**ND(2)	Y(2)**/**N(2)	Y(0)**/**N(2)	Y(0)**/**N(5)/ND(3)	Y(0)**/**N(5)/ND(3)
BMD *Z*‐score (lumbar DXA)	[+8.1 (2y), +11.9 (5y)] [+8 (20 m), +7.1 (3.4y), +6.4 (4.5y), +5.1 (5.5y)] [+0.6 (5.5y?)]				
Others	Delayed closure of fontanelle (2), Macrocephaly (1), Melanocytic nevi	Craniosynostosis, intracranial extramedullary hematopoiesis		Brain malformations, calcifying leukoencephalopathy, epilepsy	Acroosteolysis, osteonecrosis/osteomyelitis of jaw

N = no; NA = not applicable; ND = not defined; Y = yes.

^a^ Splice donor site mutations.

^b^ Osteomyelitis.

**Fig. 1:**
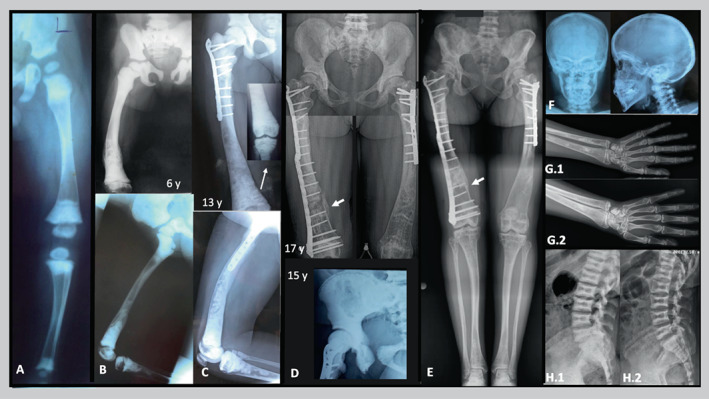
Patient 1. Evolution of radiographic findings at increasing age. (*A*) In early childhood the pelvis and metaphyses and epiphyses of the lower extremities are densely osteosclerotic. (*B*) At age 6 years, osteosclerosis is more diffuse and also apparent in the lower spine. Right femoral tubulation error and transverse healing fracture are apparent distally. (*C*) At age 13 years, the right femur shows coxa vara, diffuse but patchy osteosclerosis, loss of cortical and medullary distinction, plate and screw fixation of a proximal fracture, removal of distal femoral hardware, and metaphyseal and distal diaphyseal expansion. The right knee is shown as an inset (arrow). (*D*) At age 15 years, the pelvis and femoral heads are densely osteosclerotic, but less so centrally, giving a “bone‐in‐bone” appearance. At age 17 years, the femora are becoming osteopenic but with coarse trabeculation and focal osteosclerosis, have hardware fixation of a fracture, an unbridged distal right fracture (arrow) that persisted at age 18 years (not shown), and distal metadiaphyseal expansion. (*E*) At age 23 years, standing radiograph shows improvements including the pelvis and lower extremities with decreased osteosclerosis and tubulation error, improved mineralization, and a solidly bridged fracture (arrow). There is expanded metadiaphyseal bone in the distal femurs and tibias, overall osteopenia, focal osteosclerosis, thick trabeculae, and bowing. (*F*) Anteroposterior and lateral views of the skull at age 12 years reveal mild diploic thickening and diffuse osteosclerosis that includes the base, orbits, facial bones, and cervical spine. (*G.1*) At age 17 years, the distal forearms have metadiaphyseal expansion and focal osteosclerosis. The carpals show peripheral osteosclerotic ringing, metacarpals and phalanges patchy osteosclerosis, and metacarpals some error of tubulation. (*G.2*) At age 23 years; the tubulation errors are more prominent, but with improvement in the patchy osteosclerosis. (*H.1*) Between ages 17‐1/2 years and 22 years (*H.2*), the vertebral endplate sclerosis (“sandwich vertebrae”) is less, matching the BMD *Z*‐scores that decreased from +8.7 to +7. The vertebral body flattening and decreasing osteosclerosis apparent in the later views of the pelvis were little changed (not shown).

Referral at age 17 years was for recurring fractures. Her height was 146 cm (−2.8 standard deviation [SD]), arm span 159 cm; and arm span‐height difference 12.7 cm (Nl −6.3 to +6.5). She weighed 53 kg (−0.7 SD). Mid‐parenteral height was 156 cm, and her healthy 30‐year‐old and 28‐year‐old sisters were 158 cm and 161 cm tall, respectively. Disproportionate short stature included a short trunk, but prior femoral fractures explained her nearly normal upper‐lower segment ratio of 0.8 (Nl 0.88–1.1). Mild prognathism, prominent nose, short philtrum, small maxillary lateral incisors, and broad shoulders were apparent. At age 18 years, hypertrichosis/hirsutism (Ferriman‐Gallwey score = 15) was without hyperandrogenemia or menstrual irregularity. Audiology and fundus and visual field examinations were normal. DSS became suspected when her radiographs revealed marked metaphyseal sclerosis that became increasingly generalized from age 1–15 years (Fig. 1A‐D). Patchy diaphyseal osteosclerosis, platyspondyly with “sandwich vertebrae,” and thickening and osteosclerosis of her calvarium were striking (Fig. 1F‐H). Then, accompanying the decline in spinal aBMD, long bone osteosclerosis diminished, leaving scarce and lacy sclerotic areas within an osteopenic background. At age 23 years, her long bones were under‐modeled and osteopenic, whereas fractures had healed with osteosclerosis (Fig. 1E).

#### Patient 2

**Fig. 2:**
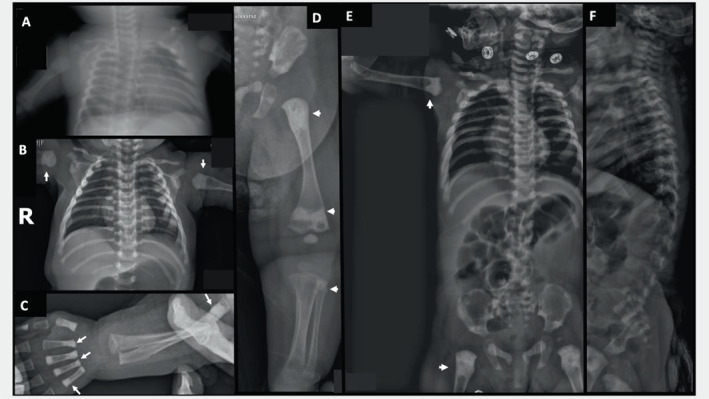
Patient 2. Radiographic findings during infancy. (*A*) At age 19 days, anteroposterior radiograph of the chest shows minimal osteosclerosis. (*B*) At age 2 months, marked metaphyseal osteosclerosis affects the proximal humeri (arrows). The clavicles, ribs, scapulae, and spine are osteosclerotic. (*C*) At age 2 months, metaphyseal osteosclerosis had faded in the distal left radius and ulna, possibly due to healing fractures there, but is apparent in four metacarpals (arrows) and proximal ulna (arrow). (*D*) At age 2 months, the dense metaphyseal osteosclerosis is no longer prominent at the provisional zone of calcification in the distal femur, and dense metaphyseal osteosclerosis is absent at the ends of the tibia (arrows). The proximal tibia and fibula (arrow) show healing fractures. Osteosclerosis is peripheral in the iliac bone and focal in the ischium and pubis. (*E*,*F*) At age 6 months, anteroposterior and lateral views of the spine demonstrate thick and osteosclerotic ribs and osteosclerosis of the clavicles, scapulae, proximal humeri, spine (especially the cervical region), and pelvis.

Patient 2 was homozygous for a different novel *SLC29A3* mutation. Her parents were second‐degree cousins. Fetal ultrasound indicated no antenatal fractures or deformities. Pregnancy and delivery were uneventful and birth weight was 3270 g. At age 2 months, a fibular fracture prompted referral for “osteogenesis imperfecta.” Height, weight, and head circumference were 57.5 cm (−0.22 SD), 4.7 kg (−0.97 SD), and 38.5 cm (−0.64 SD), respectively. Frontal bossing, open anterior (3 × 4 cm) and posterior (0.5 × 0.5 cm) fontanelles, gray‐blue sclera, mild hypertrichosis, and restricted elbow extension were present. Growth was good with appropriate neurodevelopmental milestones at age 6 months. Audiology, ophthalmology, metabolic and heavy metal screening, and BTMs were normal except for elevated urinary DPD (Table 2). Metaphyseal osteosclerosis was not apparent at age 19 days, but at age 2 months was documented in her femurs, proximal humeri, and metacarpals when referred to our clinic. At age 6 months, platyspondyly and “sandwich vertebrae” were absent on spinal radiographs (Fig. 2A‐F).

#### Patient 3

**Fig. 3:**
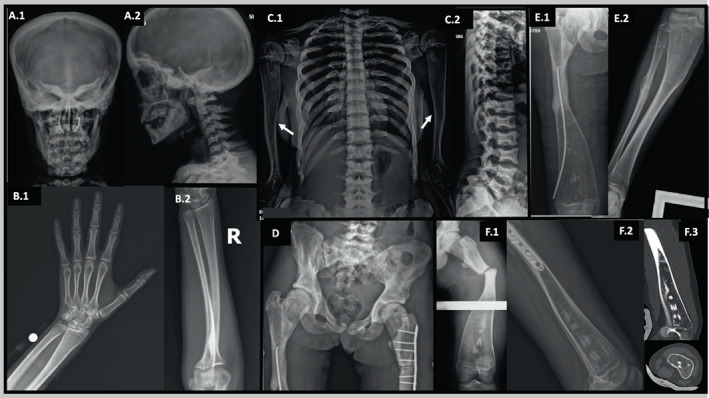
Patient 3. Radiographic findings at age 17 years. (*A.1*,*A.2*) Anteroposterior and lateral views of the skull show thick diploic space and diffuse osteosclerosis including the base, orbits with sphenoid wings, petrous bones, maxilla, mandible, and cervical spine. (*B.1*,*B.2*) Bones of the right hand and forearm are mildly undertubulated, but not osteosclerotic. (*C.1*,*C.2*) Osteosclerosis is marked in the wide ribs and present in portions of the clavicles. The vertebrae are sclerotic and flattened with coarse trabeculae and concavities of their superior and inferior margins are associated with disk expansion. Marked medullary cavity expansion distorts the proximal humeri showing cortical thinning and small focal metaphyseal osteosclerosis (arrows). (*D*) Anteroposterior view of the pelvis and proximal femurs shows diffuse patchy osteosclerosis and sclerotic rings paralleling the iliac crests, asymmetry, osteosclerotic femoral heads, wide femoral necks and trochanteric regions, and coxa valga. On the right, there is osteosclerosis and obliteration of the medullary cavity below the trochanters, a lateral cortical fracture line and intramedullary nail, and mild acetabular protrusio. On the left, a proximal femoral transverse fracture with plate‐and‐screw fixation lacks solid bridging, although new bone is medial to the fracture. (*E.1*,*E.2*) The right femur and tibia/fibula show marked expansion of the medullary cavities. The femur and tibia demonstrated cortical thinning at their ends and mid‐portion narrowing. The narrow mid femur has no medullary cavity. Bone atrophy has occurred proximal to the superior end of the femoral nail. A healing mid‐shaft fibular fracture is apparent. (*F*) The left femur shows marked medullary expansion and cortical thinning, focal areas of osteosclerosis, and an acute transverse fracture through its narrow mid portion (*F.1*), fixed internally with plate and screws (*F.2*). Computed tomography demonstrates the markedly thin cortex, expanded medullary cavity, and thin trabeculae (*F.3*).

Patient 3, 21 years old, harbored a unique homozygous mutation in *TNFRSF11A*. Her parents were consanguineous. At age 2 years, nystagmus, pale optic disks, and increased retinal vessel tortuosity were noted. She presented to us at age 10 years with short stature; height 121 cm (−3.3 SD) and weight 23.6 kg (−2.2 SD). Body proportions were normal (upper‐lower segment ratio 1.04 [Nl 0.86–1.08], and arm span/height difference 3.4 cm [Nl −7.8 to +6.1]). Family members were not short, and reportedly without heritable diseases. Pycnodysostosis was suspected because her facial dysmorphism included proptosis, beaked nose, malar hypoplasia, and short philtrum. Nystagmus had been first noticed at age 2 months, and was present at age 18 years when cranial and orbital tomography showed bilateral proptosis and thickening of optic nerve sheet indicating optic canal narrowing. Poor oral hygiene, crowded teeth, and multiple caries were present. Immunoglobulin levels were not measured because there had been no recurrent infections. Femoral fractures, three requiring surgery, began at age 16 years. Adult height was 138 cm (−4.2 SD) with mid‐parenteral height 154 cm (−1.55 SD). Arm span was 151 cm, arm span/height difference 12.2 cm (Nl −6.3 to +6.5 cm), and upper‐lower segment ratio 0.88 (Nl 0.88–1.1) showing that her short stature principally reflected her short trunk. Cranial bones and ribs were osteosclerotic (Fig. 3A,C1). Platyspondyly with vertebral endplate osteosclerosis was present (Fig. 3,C2). Long bones featured osteopenia, thin cortices, and wide metaphyses (Fig. 3E‐F). Pycnodysostosis seemed unlikely because hand radiographs lacked osteosclerosis or acroosteolysis, and her osteopenic long bones featured localized osteosclerosis consistent with DSS (Fig. 3B,E). DXA of L_1_–L_4_ revealed elevated aBMD and *Z*‐scores increasing with age; +8.1 at 18 years, +9.3 at 19 years, and +10.7 at 21 years. BTMs spanning 17 to 21 years old were normal (Table 2). In 2017, we identified her homozygous *TNFRSF11A* mutation in our research laboratory (see Mutation analyses). Then, in 2018, Guo and colleagues^(^ ^20^ ^)^ reported her the first example of DSS from *TNFRSF11A* mutation.

#### Patient 4

Patient 4, 11 years old, revealed in our research laboratory no mutation in any of the five genes (*SLC29A3*, *TNFRSF11A*, *LRRK1*, *TCIRG1*, and *CSF1R*) that had come to be associated with DSS (see Discussion). His parents were unrelated. He weighed 2.2 kg when delivered at 36 weeks gestation to a 25‐year‐old woman who had noticed diminished fetal movement during the last week of her pregnancy. Intensive care for 10 days included respiratory support for 3 days. At age 6 months, slow motor development was reported. At age 14 months, inborn‐error‐of‐metabolism studies were negative, and OMD was considered when radiographs showed metaphyseal osteosclerosis. Remarkably, at age 27 months, fever, cough, and vomiting accompanied unexplained severe hypercalcemia (19.8 mg/dL [Nl 8.8–10.6]) with physiologically suppressed serum parathyroid hormone (PTH), hypophosphatemia, low‐normal ALP, and normal 25‐hydroxyvitamin D (Table 2). Intravenous hydration and pamidronate (1 mg/kg/day for 3 days) corrected the hypercalcemia, which did not recur. Frequent respiratory tract infections required four hospitalizations for pneumonia. Early developmental milestones were slightly delayed, but by age 2 years global motor retardation was apparent, and by age 4.5 years he could not sit, walk, or pronounce words. Cranial magnetic resonance imaging (MRI) was reportedly normal except for mild cortical atrophy.

**Fig. 4:**
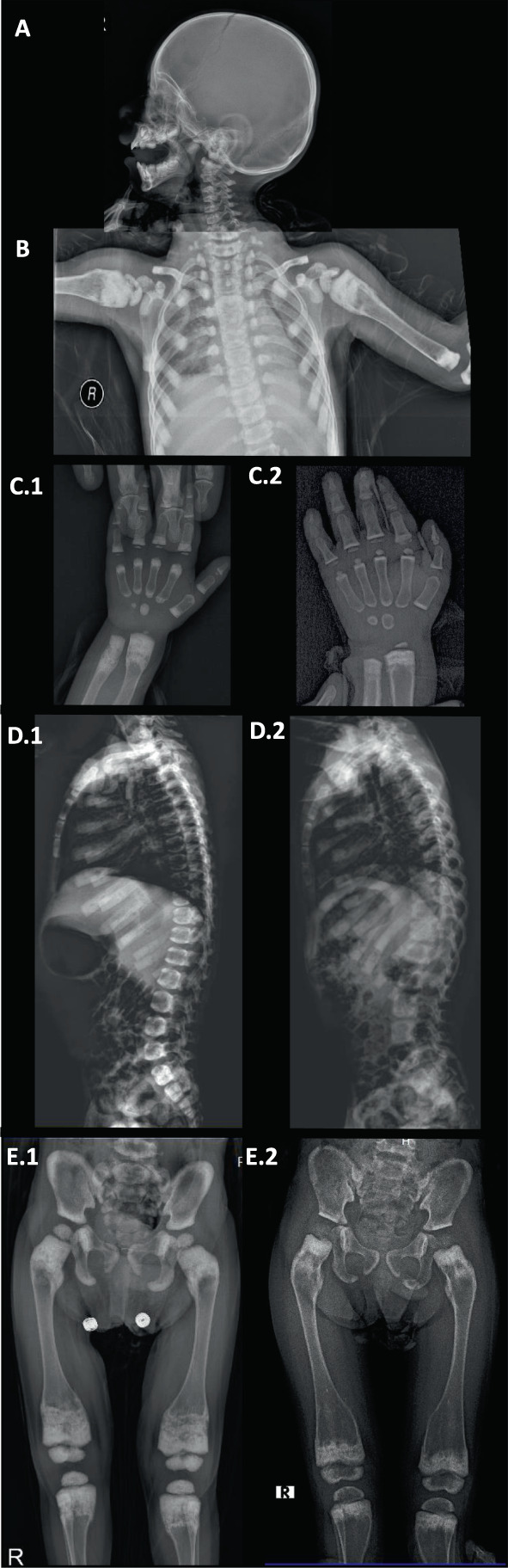
Patient 4. Radiographic findings in early childhood. (*A*) At age 2 years, the skull has slightly increased vertical diameter. (*B*) There is marked metadiaphyseal osteosclerosis and widening of the proximal humeri, marked osteosclerosis and some widening of the ribs, and osteosclerosis of the spine, clavicles, and scapulae. (*C.1*,*C.2*) Between ages 2‐1/3 and 4‐1/3 years, osteosclerosis has decreased in the metaphyses and carpals in the hands and wrists, and metadiaphyses in the radius and ulna. Metadiaphyseal widening has increased. (*D.1*,*D.2,E.1*,*E.2*) Osteosclerosis has decreased in the pelvis and lateral spine and metadiaphyses of the femurs and proximal tibias and fibulas. Metadiaphyseal expansion and cortical thinning are developing in the tubular bones.

When referred to us at age 28 months, his height, weight, and head circumference *Z*‐scores were −1.8, −4.2, and −3.0, respectively. Dysmorphic facial features included a wide forehead, triangular face with open mouth, high‐arched palate, yellow teeth, short nose with prominent nasal tip, long philtrum, mild hypoplasia of alae nasi, nasal obstruction, rigid pinnae, almond‐shaped eyes with gray sclera, and laterally anteverted palpebral fissures (Kabuki makeup syndrome–like). Skeletal deformity was absent, but spasticity and atrophic muscles were present. Skin changes of DSS, such as red rashes, were present. Oral candidiasis was detected although serum immunoglobulin levels, immune phenotyping, nitroblue tetrazolium (NBT) testing, metabolic screening (including phosphoethanolamine for hypophosphatasia), blood gases, and lead level were normal. Adenoidectomy was performed at age 3.5 years. Scoliosis persisted from age 4.5 years. BTMs were unremarkable except for low OCN, but assayed after pamidronate treatment (Table 2). At age 11 years, hospitalizations for recurrent infections had ceased. He had not fractured. Radiographs revealed wide sclerotic metaphyses with osteopenic diaphyses, wide sclerotic ribs, and “sandwich vertebrae” but without platyspondyly (Fig. 4B‐E). Osteosclerotic areas had receded since early childhood, but with increasingly osteopenic widened bones with thin cortices (Fig. 4C1‐E1 to C2‐E2). His spinal aBMD was unchanged from 0.54 to 0.52 g/cm^2^, therefore generating *Z*‐scores that decreased from +3.02 to +0.43 as he grew and aged.

### Mutation analyses

Mutation analyses were performed for patients 1, 3, and 4 in our research laboratory at Washington University School of Medicine, St. Louis, MO, USA. Written consent was obtained as approved by the Marmara University Medical Faculty Research Ethics Committee (MAR‐Y‐09.2021‐623) Istanbul, Turkey in accordance with the 1964 Helsinki declaration and its later amendments. Coding exons and exon‐intron boundaries of *SLC29A3* were Sanger sequenced using methods we had developed.^(^ ^4^, ^5^ ^)^ When no *SLC29A3* mutation was identified (patients 3 and 4), Ion Torrent (Thermo Fisher Scientific) next‐generation sequencing (NGS) examined 35 genes that: (i) cause osteosclerotic disorders, (ii) condition skeletal remodeling, or (iii) reflect mouse models featuring elevated bone mass: ie, *TNFRSF11A (RANK)*, *TNFRSF11B (OPG)*, *TNFSF11 (RANKL)*, *VCP*, *SQSTM1*, *TGFB1*, *IFITM5*, *MAFB*, *CSF1*, *CSF1R*, *TRAF6*, *RELA*, *RELB*, *REL*, *NFKB1*, *NFKB2*, *TFEB*, *CA2*, *CLCN7*, *CTSK (CATHEPSIN K)*, *OSTM1*, *PLEKHM1*, *TCIRG1*, *SOST*, *SLC29A3*, *LRP4*, *LRP5*, *LRP6*, *SNX10*, *FAM20C*, *FAM123B (AMER1)*, *TYROBP*, *LEMD3*, *DLX3*, and *PTDSS1*.^(^ ^4^, ^5^ ^)^ Variants were then verified by Sanger sequencing. For patient 4, *LRRK1* was Sanger sequenced using primers we designed (sequences available on request). At least 10 bases of intronic DNA sequence at each exon/intron boundary were sequenced to identify potential messenger RNA (mRNA) splice site mutations. Patient 2 was studied at Marmara University School of Medicine where clinical exome sequencing was performed using SOPHIA Clinical Exome Solution V2 via Illumina Nextseq 550 platform (Illumina, San Diego, CA, USA). Twenty‐seven genes: *TNFRSF11A (RANK)*, *TNFRSF11B (OPG)*, *VCP*, *SQSTM1*, *TGFB1*, *IFITM5*, *MAFB*, *CSF1*, *CSF1R*, *TRAF6*, *NFKB2*, *CA2*, *CLCN7*, *CTSK (CATHEPSIN K)*, *OSTM1*, *TCIRG1*, *SOST*, *SLC29A3*, *LRP4*, *LRP5*, *LRP6*, *SNX10*, *FAM20C*, *FAM123B (AMER1)*, *TYROBP*, *LEMD3*, and *DLX3* were filtered. SOPHIA DDM‐V4 analyzed the data. For segregation analysis, we used the Illumina MiSeq platform.

## Results

### Mutational analyses

#### Patient 1

Sanger sequencing of *SLC29A3* revealed a novel homozygous 18‐basepair (bp) duplication in exon 3 (c.303_320dup, p.102_107dupYFESYL) predicting in‐frame tandem duplication of six amino acid residues (Fig. S1). Her consanguineous parents and one of two healthy sisters were heterozygous for this mutation.

#### Patient 2

NGS of *SLC29A3* revealed a novel homozygous nonsense mutation (c.1284C>G, p.Tyr428*) that was heterozygous in her consanguineous parents (Fig. S2).

#### Patient 3

All exons and adjacent mRNA splice sites of *SLC29A3* were intact. In 2017, our NGS revealed a novel homozygous splice donor site variant (c.616+3A>G) in intron 6 of *TNFRSF11A* (Fig. S3). This variant was heterozygous in her mother; the father was not available for study. The following year, Guo and colleagues^(^ ^20^ ^)^ confirmed this mutation and proposed it caused DSS (see Discussion).

#### Patient 4

Our mutation analysis was negative for *SLC29A3*, *TNFRSF11A*, *TCIRG1*, *LRRK1*, and *CSF1R* and the OPT‐associated genes *CLCN7*, *TNFSF11*, *CA2*, *OSTM1*, *PLEKHM1*, and *SNX10*. This included sequencing all coding exons and at least 10 bases of intronic DNA at exon/intron junctions while recognizing that Guo and colleagues^(^ ^22^ ^)^ in 2019 had reported deep intronic mutations in *CSF1R* causing DSS, resulting in “abnormal inclusion of intron sequences in the mRNA.” Additional methodologies to identify this type of mutation (eg, reverse transcription‐polymerase chain reaction using patient‐derived RNA) could not be undertaken for this patient.

### DSS phenotype of our patients

Our four patients' clinical, radiographic, and genetic findings indicating DSS (Table 1) and assessments of mineral and skeletal metabolism (Table S1) were then compared with published reports of DSS.

### Literature evaluation

The genotype/phenotype spectrum of DSS was assessed from the literature using Medline, Embase, Scobus, Web of Science, bone abstracts and Mendeley and was then summarized (Table 2).

## Discussion

From its first reporting in 1934^(^ ^1^ ^)^ until evidence of genetic heterogeneity in 2013,^(^ ^12^ ^)^ DSS seemed a distinctive Mendelian OPT.^(^ ^1^, ^2^, ^6^, ^7^, ^8^, ^9^, ^10^, ^24^ ^)^ In 2010, we described in a girl with classic features of DSS absence of OCs and unresorbed calcified primary spongiosa emblematic of OPT.^(^ ^4^ ^)^ In 2012, we reported that she and a similarly affected girl manifested impaired osteoclastogenesis and OC action and discovered they harbored biallelic *SLC29A3* mutations.^(^ ^5^ ^)^ In 2015, The International Skeletal Dysplasia Registry^(^ ^25^ ^)^ regarded DSS as an OPT‐like disorder.^(^ ^2^, ^6^, ^7^, ^8^, ^9^, ^10^, ^24^, ^26^, ^27^, ^28^, ^29^, ^30^ ^)^

Although osteosclerosis and impaired bone modeling (ie, “undertubulation”) characterize the OPTs including DSS, the platyspondyly and spontaneous resolution of the osteosclerosis of DSS do not.^(^ ^6^, ^10^ ^)^ DSS features acquired platyspondyly and osteosclerosis (especially in the appendicular skeleton), but patchy osteosclerosis later on, and osteopenia in adulthood.^(^ ^6^, ^10^, ^11^ ^)^ Hence, DSS has a particularly complex and enigmatic pathogenesis. The changing skeletal phenotype can go undocumented or unappreciated and therefore undiagnosed. By 2022, among 23 individuals with mutational analysis from 45 with the DSS phenotype,^(^ ^6^, ^7^, ^8^, ^9^, ^24^, ^26^, ^27^, ^28^, ^29^, ^30^ ^)^ six, four, two, and 11 had defects of *SLC29A3*, *TNFRSF11A*, *TCIRG1*, or *CSF1R*, respectively.^(^ ^5^, ^11^, ^20^, ^21^, ^22^, ^23^, ^31^, ^32^, ^33^, ^34^, ^35^, ^36^ ^)^ The heterogeneity among the clinical and radiological presentations, complications, and prognoses for each etiology is as follows.

### Our patients' DSS

Patient 1, our oldest at 23 years old, provided almost a 20‐year follow‐up, whereas patient 2 is the youngest reported with DSS (Tables 1 and 2). Short stature was invariable^(^ ^2^, ^4^, ^5^, ^6^, ^7^, ^10^, ^20^, ^21^, ^31^, ^32^, ^33^, ^34^ ^)^ whereas arm length was unaffected. Disproportionate dwarfism featuring a short trunk became a constant finding by adulthood despite recurrent femoral fracturing. The body proportions of patient 3 changed from age 10 to 17 years, highlighting her progressive trunk shortening. However, platyspondyly with a shortened trunk was not evident early on in our youngest patients 2 and 4. Patient 3's fractures began relatively late; ie, in adolescence.

In DSS, discoloration and delayed eruption of teeth, dentition embedded into the gum, frequent caries, and mandibular osteomyelitis have been reported.^(^ ^7^, ^9^, ^10^, ^11^, ^16^, ^20^, ^24^, ^26^, ^27^, ^28^ ^)^ Patients 1 and 2 had delayed shedding of deciduous teeth followed by delayed eruption of permanent teeth, or were too young for teething, respectively. Patient 3 suffered tooth decay. Patient 4 had discolored teeth. Only patient 3, homozygous for a *TNFRSF11A* mutation, had nystagmus due to optic atrophy from narrowed optic canals related to the osteosclerosis of her cranial bones.

Herein, we found metaphyseal osteosclerosis in early childhood. However, patient 2 demonstrated it might not be present neonatally, yet appear by age 2 months. For patient 1, followed to age 22 years, metaphyseal osteosclerosis extended as she grew, but mid‐diaphyseal radiolucency persisted up to age 11 years. Then, patchy osteosclerosis with sparse radiolucency involved the diaphyses. At older ages, radiolucent areas predominated, especially after physeal fusion and cessation of growth. Axial osteosclerosis, including of the ribs, pelvis, and vertebrae in patients 1, 3, and 4 was a more constant finding. However, in patient 1 it was decreased during adulthood. In patient 3, osteosclerosis increased steadily with better demarcated focal osteosclerosis, especially mid‐diaphyseal, and fracture‐prone dense bone within osteopenic long bones. Perhaps absence of apparent metaphyseal sclerosis together with unaffected hand and forearm bones indicates DSS due to *TNFRSF11A* mutation. Patient 4's metaphyseal and marginal osteosclerosis faded by age 4 years, leaving severely osteopenic long bones. Resolution of osteosclerosis can occur in the carbonic anhydrase II deficiency form of OPT (OMIM, type 3),^(^ ^3^ ^)^ perhaps because its unique metabolic acidosis leaches mineral from bone.^(^ ^37^ ^)^ Patient 4 manifested unexplained marked hypercalcemia once, but was without a *CA2* mutation or acidosis. Although no fractures had been detected, perhaps his hypercalcemia reflected his immobility. Hypercalcemia is not a feature of the OPTs unless following restoration of OC action by marrow cell transplantation.^(^ ^38^, ^39^ ^)^ In our initial patient harboring *SLC29A3* mutations,^(^ ^4^, ^5^ ^)^ serum ionized calcium was high‐normal at approximately 1 year old, but decreased after excessive dietary calcium was corrected.^(^ ^4^ ^)^ In DSS, low serum PTH levels can occur,^(^ ^9^, ^10^ ^)^ suggesting mineral homeostasis is impacted. Hypocalcemia from decreased bone resorption and diminished gastrointestinal absorption of calcium underlies “osteopetrorickets” from *TCIRG1* deactivation.^(^ ^38^, ^40^ ^)^

Slow fracture healing can occur in DSS,^(^ ^4^ ^)^ as in other OPTs. Indeed, quiescent bone remodeling was suggested in our four patients by low serum OCN levels and low‐normal ALP activity. Patients 1 and 2 had elevated urinary DPD, a marker of bone resorption, whereas serum CTX levels were normal. However, BTMs were inconsistent among our four patients, perhaps reflecting their genetic heterogeneity for DSS as well as evolving skeletal phenotype.

### *SLC29A3*‐associated DSS

The two unrelated girls we reported in 2012 with DSS were compound heterozygous (c.607T>C, p.Ser203Pro; c.1157G>A, p.Arg386Gln) and homozygous (c.1346C>G, p.Thr449Arg) for *SLC29A3* mutation.^(^ ^5^ ^)^ The former had Turkish heritage.^(^ ^4^ ^)^ Their few OCs formed from peripheral blood weakly demineralized a crystalline calcium‐phosphate surface.^(^ ^4^, ^5^ ^)^ Low serum tartrate‐resistant acid phosphatase (TRAP) matched a paucity of OCs.^(^ ^4^ ^)^ Therefore, OC formation and function in DSS can become impaired and increase bone mass, but then apparently recover sufficiently to resorb calcified primary spongiosa and osteosclerosis. Currently, however, OMIM^(^ ^3^ ^)^ does not mention our 2010 report of OC‐poor OPT in DSS^(^ ^4^ ^)^ and considers the two patients examples of “H syndrome” or “histiocytosis‐lymphadenopathy plus syndrome” (602782), which features biallelic *SLC29A3* mutation causing short stature (but not fracturing), histiocytosis, and lymphadenopathy with or without cutaneous, cardiac, and/or endocrine features (insulin‐dependent diabetes mellitus, hypogonadism), joint contractures, and/or deafness. Now, six individuals have been reported to have DSS from *SLC29A3* mutation.^(^ ^5^, ^21^, ^31^, ^32^ ^)^

### *TNFRSF11A*‐associated DSS

Beginning in 2008,^(^ ^18^ ^)^ homozygous mutation of *TNFRSF11A* encoding RANK was reported to cause “OC‐poor” OPT. About 20 examples are published.^(^ ^18^, ^39^, ^40^, ^41^, ^42^, ^43^, ^44^, ^45^, ^46^ ^)^ However, in 2018 Guo and colleagues^(^ ^20^ ^)^ specified that our patient 3 had DSS. Now, they attribute the DSS phenotype to four individuals with biallelic loss‐of‐function defects in *TNFRSF11A*.^(^ ^11^, ^20^, ^33^, ^34^ ^)^ Splice site mutations of *TNFRSF11A*, exemplified by our patient 3, that truncate or extend the encoded RANK protein can cause DSS^(^ ^11^, ^20^ ^)^ whereas “DSS” from *TNFRSF11A* missense mutation p.R129C^(^ ^34^ ^)^ featured diffuse osteosclerosis and extramedullary hematopoiesis consistent with a severe OPT. *TNFRSF11A* mutations that alter N‐terminal folding of the encoded RANK and compromise its interaction with RANKL^(^ ^18^ ^)^ cause OPT, whereas some functional RANK seems to cause DSS. Nevertheless, the skeletal phenotype of the severe OPT, can also improve with aging.^(^ ^11^ ^)^ The eldest person (age 59 years) reported with biallelic *TNFRSF11A* mutations had severely osteopenic long bones.^(^ ^11^ ^)^

### *TCIRG1*‐associated DSS

In 2018, in two siblings, compound heterozygosity of *TCIRG1* (c.117+4A>C with c.2380_2381delCT, p.A796fs*34) reportedly caused metaphyseal sclerosis typical of DSS.^(^ ^21^ ^)^ *TCIRG1* encodes a component of the vacuolar proton (H+) pump, and biallelic mutations thereby cause “osteopetrorickets” from an abundance of nonfunctional OCs together with impaired gastrointestinal absorption of calcium due to hypochlorhydria.^(^ ^38^, ^40^ ^)^ The OPT from biallelic *TCIRG1* defects is “OC‐rich” and treatable by hematopoietic stem cell transplantation that generates functional OCs.^(^ ^19^, ^38^, ^40^, ^41^ ^)^ The *TCIRG1* splice site mutation at the beginning of this paragraph (c.117+4A>C), is homologous to splice site mutation c.117+4A>T reported in 2000 to cause OPT^(^ ^47^ ^)^ in a Turkish patient. When this mutation accompanied p.A796fs*34 above, the phenotype was milder.

### *LRRK1*‐associated osteosclerotic metaphyseal dysplasia

The eight individuals reported to date with “osteosclerotic metaphyseal dysplasia” (OMIM % 615198)^(^ ^3^ ^)^ harbored homozygous defects of *LRRK1*.^(^ ^13^, ^14^, ^15^, ^16^, ^17^ ^)^ Their clinical and radiographic features of DSS resemble those attributable to *SLC29A3* or *TCIRG1* mutation, including fractures without severe extramedullary hematopoiesis or short stature.^(^ ^13^, ^14^, ^15^, ^16^, ^17^ ^)^ Radiological features include metaphyseal and vertebral sclerosis with mildly under‐modeled long bones that may evolve, including diminishing metaphyseal sclerosis.^(^ ^14^ ^)^ However, there is no prominent platyspondyly.

### *CSF1R*‐associated DSS‐Pyle disease spectrum

The “DSS‐Pyle disease spectrum” features generalized osteosclerosis and irregular dysplastic metaphyses associated with neonatal and infant lethality from leukoencephalopathy, intracranial calcification, Dandy‐Walker malformation, cystic dilation of the posterior fossa and ventricles, and agenesis of the corpus callosum.^(^ ^48^, ^49^ ^)^ In 2017, in first‐cousin carrier parents, heterozygous mutation (p.Y540*) of *CSF1R* was detected.^(^ ^48^ ^)^ Biallelic *CSF1R* mutations were identified by Guo and colleagues^(^ ^22^ ^)^ and Kındış and colleagues^(^ ^35^ ^)^ in 2019 and 2021, respectively. The 2019 report^(^ ^49^ ^)^ concerned a homozygous splice acceptor site mutation (c.1754−1G>C), published back‐to‐back by Guo and colleagues.^(^ ^22^ ^)^ However, these two reports describe an osteosclerotic phenotype atypical for DSS.^(^ ^48^, ^49^ ^)^ In fact, biallelic *CSF1R* defects do not always have a bone phenotype; eg, the second case, harboring p.H643Q, reported in 2019 by Oosterhof and colleagues,^(^ ^49^ ^)^ and two siblings with the p.T833M mutation.^(^ ^50^ ^)^ Monoallelic *CSF1R* mutation underlies hereditary diffuse leukoencephalopathy with spheroids (HDLS)—a rapidly progressive lethal neurodegenerative disease of adults that features cerebral white matter, behavioral, cognitive, and motor changes as well as dementia.^(^ ^51^ ^)^ However, no bone changes were reported in 2021 by Guo and Ikegawa from a relatively large experience.^(^ ^52^ ^)^ Typical HDLS has occurred with “sclerosing skeletal dysplasia”, with undertubulation and flaring of the metaphyses of most of the tubular long bones and fish‐shaped vertebrate.^(^ ^36^ ^)^ An osteosclerotic adolescent boy with HDLS reported in 2020 by Breningstall and Asis^(^ ^36^ ^)^ was heterozygous for *CSF1R* p.Q481*. His radiographs, kindly provided to us by Dr. G.N. Breningstall (unpublished), showed platyspondyly and osteopenic long bones with Erlenmeyer flask deformity and focal osteosclerosis. His *CSF1R* defect was compound heterozygous in a patient with “brain abnormalities, neurodegeneration, and dysosteosclerosis” (BANDDOS: OMIM # 618476).^(^ ^22^ ^)^ The father, who was heterozygous, had mild cortical hyperostosis and normal cranial tomography, yet short‐term memory loss from age 70 years, and parenchymal calcification were detected in the 76‐year‐old paternal grandfather, who was also heterozygous.^(^ ^22^ ^)^

Our patient 4, lacking identification of a causal gene, uniquely suffered severe mental/motor retardation, infections, and one episode of severe unexplained hypercalcemia. In DSS, infections have been frequent without any detectable immune defect.^(^ ^4^, ^5^ ^)^ Neurodevelopmental delay, macrocephaly, seizures, intracranial calcification, and delayed myelination are features of the DSS‐Pyle disease spectrum.^(^ ^7^, ^9^, ^22^, ^35^ ^)^ His cranial MRI showed a myelinization defect, but no leukoencephaly or findings of Pyle disease. Microcephaly, not macrocephaly, was present.

### DSS from *SLC29A3* versus *TNFRSF11A* mutation

The DSS phenotype associated with *SLC29A3* and *TNFRSF11A* mutations features short stature. However, fracturing sometimes with slow healing (eg, patient 1),^(^ ^5^ ^)^ begins earlier and seems more common with *SLC29A3* mutations (eg, patients 1 and 2). Metaphyseal sclerosis characteristic of DSS was not observed at different ages in three of four patients harboring *TNFRSF11A* mutations.^(^ ^11^, ^20^, ^33^, ^34^ ^)^ The exception differed from DSS because diffuse diaphyseal sclerosis ended with a small area of osteopenia and continued with the osteosclerotic metaphyses.^(^ ^33^ ^)^ *TNFRSF11A*‐associated DSS features particularly severe osteosclerosis of the axial skeleton together with optic atrophy, craniosynostosis, and extramedullary hematopoiesis.^(^ ^34^ ^)^ Contrary to DSS from defective *SLC29A3* and *TCIRG1*, mild *TNFRSF11A* mutations likely leave hand bones unaltered (eg, patient 3), whereas severely compromised RANK protein can cause marked osteosclerosis of the entire skeleton including hand bones.^(^ ^33^, ^34^ ^)^

### Conclusions

Our experience improves understanding of the clinical, radiographic, and genetic heterogeneity of the DSS phenotype. The changing radiographic hallmarks can be important to suspect and diagnose DSS, now associated with mutations of *SLC29A3*, *TNFRSF11A*, *TCIRG1*, *LRRK1*, and *CSF1R*. Further genetic heterogeneity, including an X‐linked recessive form,^(^ ^24^ ^)^ seems likely. In clinical practice, establishing the genetic basis for DSS will help understand the complications, prognosis, and treatment.

## Author Contributions

**Serap Turan:** Conceptualization; data curation; investigation; methodology; supervision; visualization; writing – original draft; writing – review and editing. **Steven Mumm:** Formal analysis; investigation; methodology; validation; writing – review and editing. **Ceren Alavanda:** Formal analysis; investigation; methodology. **Betul Sare Kaygusuz:** Data curation; investigation; visualization. **Busra Gurpinar Tosun:** Data curation; resources; visualization. **Ahmet Arman:** Data curation; investigation; methodology; validation. **Margaret Huskey:** Formal analysis; methodology; validation; visualization. **Tulay Guran:** Data curation; investigation; visualization. **Shenghui Duan:** Data curation; resources; supervision; visualization. **Abdullah Bereket:** Methodology; project administration; resources; supervision; writing – review and editing. **Michael P. Whyte:** Investigation; methodology; project administration; resources; supervision; writing – original draft; writing – review and editing.

## Conflicts of Interest

None.

### Peer Review

The peer review history for this article is available at https://publons.com/publon/10.1002/jbm4.10663.

## Supporting information


**Appendix S1:** Supplementary Information
Figs. S1–S3
Table S1

## Data Availability

All data and material will be available upon request.
