# Supplementary material for: Dysosteosclerosis: Clinical and Radiological Evolution Reflecting Genetic Heterogeneity
Source: JBMR Plus. 2022 Jul 28;6(8):e10663. doi: 10.1002/jbm4.10663 (PMC9382861; doi:10.1002/jbm4.10663)
Supplement: Supplementary file 1 — Appendix S1: Supplementary Information Figs. S1–S3 Table S1 [file JBM4-6-e10663-s001.pdf]

Supplementary Appendix

Supplementary Appendix, Figure 1:

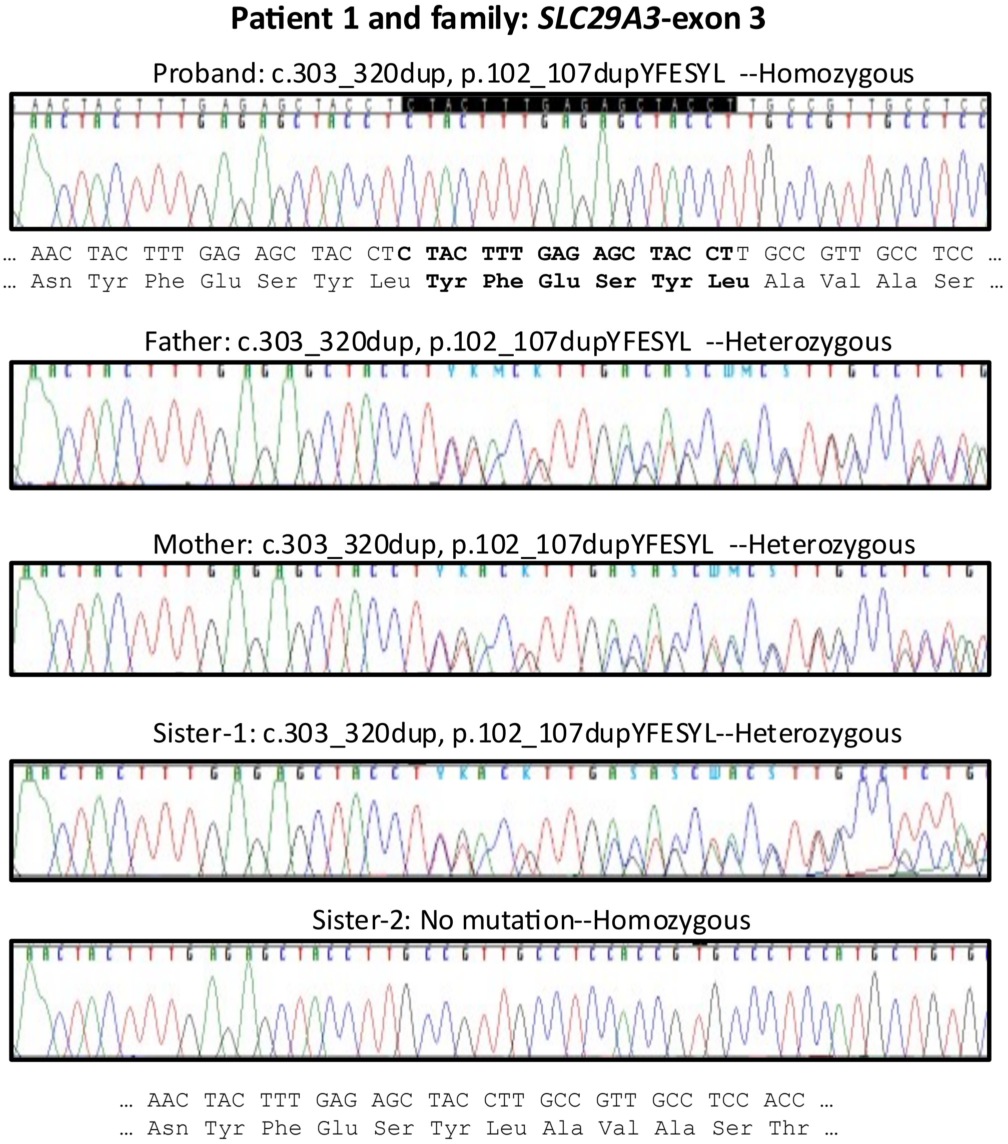

SLC29A3 Mutation In Patient 1 And Family.

Electropherograms for *SLC29A3* exon 3 are shown for the patient, parents, and two sisters. The patient's homozygous 18 bp duplication is demarcated by the black bar. Below her electropherogram are the cDNA and amino acid sequences showing the 18 bp and 6 amino acid duplications (bold). At the bottom, the wild type cDNA and amino acid sequence are depicted for sister 2 who was not a carrier of the *SLC29A3* duplication.

Supplementary Appendix, Figure 2:

Patient 2 and family: SLC29A3 - exon 6

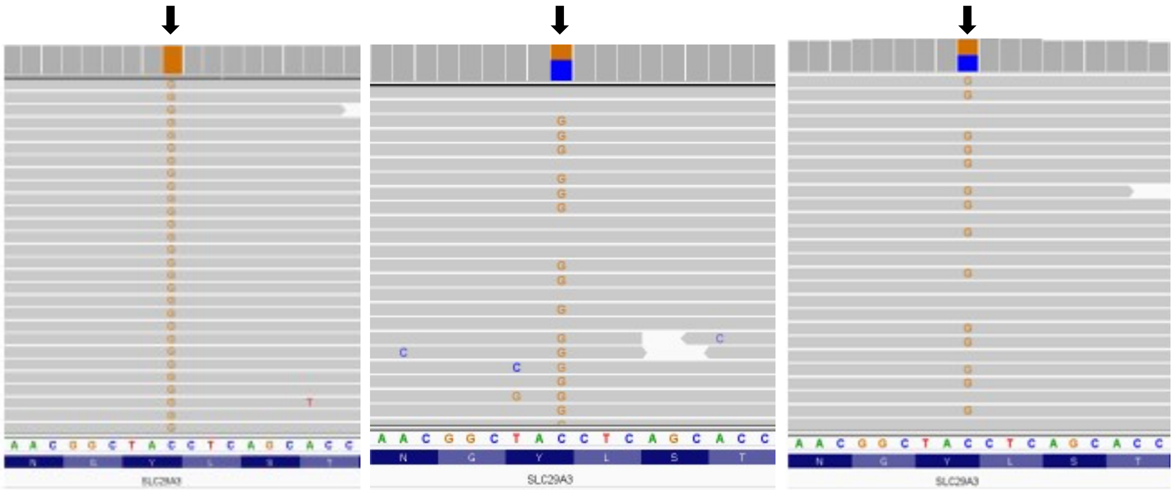

Proband:
c.1248C>G, p.Tyr428* homozygous

Father:
c.1248C>G, p.Tyr428* heterozygous

Mother:
c.1248C>G, p.Tyr428* heterozygous

SLC29A3 Mutation in Patient 2 And Parents

Integrative Genomics Viewer (IGV) visualization of *SLC29A3* exon 6 is shown for the patient and her parents. Detected c.1248C>G substitution is indicated by the arrow.

Supplementary Appendix, Figure 3:

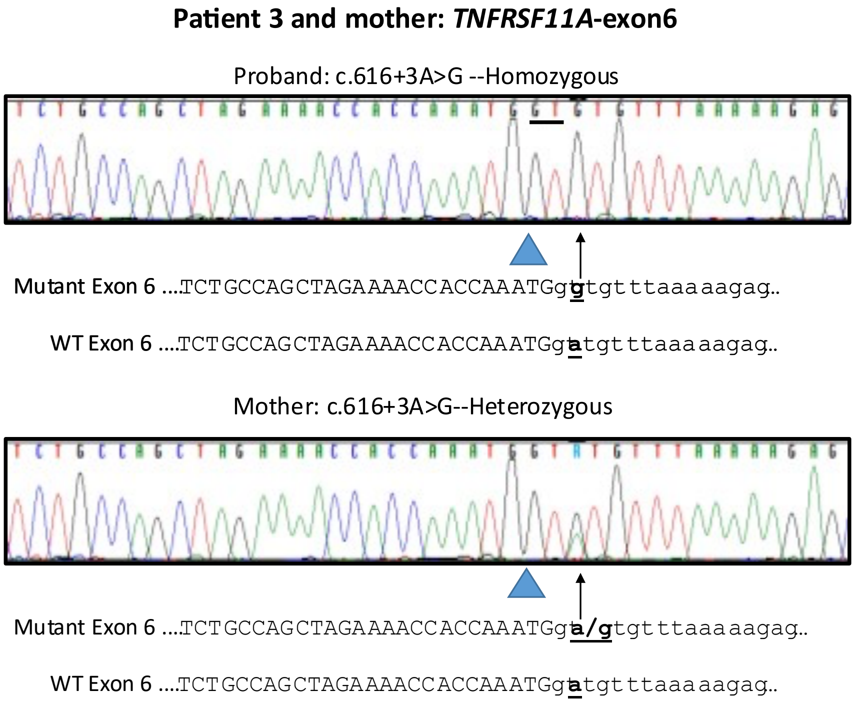

TNFRSF11A Mutation In Patient 3 And Mother.

Electropherograms for the 3′ end of exon 6 and adjacent intron are shown for patient 3 and her mother (Figure top and bottom, respectively). The blue triangle designates the exon/intron junction. The underlined GT represents the most critical bases for mRNA splicing; the mutation is adjacent. Below each electropherogram are the mutant and wild type (WT) sequences, with the mutations shown in bold.

Supplementary Appendix, Table 1. Biochemical Testing of Mineral and Skeletal Metabolism In Our Four Patients at Different Ages

**Patient**	**Patient 1**				**Patient 2**	**Patient 3**			**Patient 4**			
**Age (years)**	11	17.5	18.2	23	0.5	17.5	19	21	1.2	2	2.1[b]	4.5
**Ca (8.8-10.6 mg/dL)**	8.6	9.3	9.4	9.5	10.1	9.6	9.5	9.2	10 (9.2)	19.8	10.4 (9.5)	9.4
**I-Ca^{+2} (4.6-5.2 mg/dl)**	4.88	4.86		4.9	4.9		4.8			7.9		
**Pi (mg/dL)**	4.4<br>(4.5-5.5)	3.7<br>(2.7-4.5)	3.9<br>(2.7-4.5)	3.2<br>(2.7-4.5)	4.2	3.6<br>(2.7-4.5)	4.1	3.0<br>(2.7-4.5)	5.9 (5.1)<br>(4.5-6.7)	3.0<br>(4.5-6.7)	5.7 (4.0)<br>(4.5-5.5)	4.9<br>(4.5-5.5)
**ALP (U/L)**	171<br>(92-418)	109<br>(49-154)	104<br>(49-154)	84<br>(49-154)	325<br>(123-377)	93<br>(49-154)	108<br>(49-154)	65<br>(49-154)	135 (118)<br>(97-362)	101<br>(97-362)	118 (189)<br>(97-362)	95<br>(97-362)
**PTH (15-65 pg/ml)**	67	27	14	18.7	35.7	40	42.9	39.8	13.9 (80.4)	2.5	11.1	41
**25(OH)D**<br>**(20-100 ng/mL)**	10	10.8	6.2	15.7	41	12	20	13.5	32	66	34	24.6
**Osteocalcin (ng/mL)**	7.8<br>(12-81)	39<br>(4.5-23.7)	27.2<br>(4.5-23.7)	<2.0<br>(0.4-8.2)	68.9<br>(15.7-111.1)	9.2<br>(4.5-24)	6.1<br>(4.5-23.7)	6.2<br>(0.4-8.2)			<2.0<br>(7.4-47)	57<br>(8.4-60)
**C-Terminal Telopeptide (ng/mL)**		0.47<br>(0.025-0.57)	0.41<br>(0.025-0.57)	0.34<br>(0.025-0.57)	0.63<br>(0.43-1.7)		0.19<br>(0.025-0.57)	0.13<br>(0.025-0.57)			0.75<br>(0.54-1.71)	
**P1NP[a] (ng/mL)**				63.2<br>(20-110)	966<br>(900-1800)			20.1<br>(20-110)				
**U-Ca/Cre (mg/mg)**	0.05	0.13		0.08	0.16	0.2	0.02					0.02
**Deoxypyridinoline (nM DPD/mM Cre)**	23.7<br>(8.1-33.8)		>15<br>(1.5-8.9)	13.5<br>(1.5-8.9)	290<br>(13.4-60.4)		6.11<br>(1.5-8.9)	7.4<br>(1.5-8.9)	55<br>(13.4-60.4)			26.6<br>(13.4-60.4)

[a]Procollagen type 1 N-terminal propeptide, [b] One month after pamidronate treatment
